# Lower pre-ART intra-participant HIV-1 *pol* diversity may not be associated with virologic failure in adults

**DOI:** 10.1371/journal.pone.0190438

**Published:** 2018-01-25

**Authors:** Mary F. Kearney, Jonathan Spindler, Ann Wiegand, Wei Shao, Richard Haubrich, Sharon Riddler, Christina M. Lalama, Michael D. Hughes, John M. Coffin, John W. Mellors

**Affiliations:** 1 HIV Dynamics and Replication Program, National Cancer Institute, Frederick, MD, United States of America; 2 Advanced Biomedical Computing Center, Leidos Biomedical Research, Inc., Frederick National Laboratory for Cancer Research, Frederick, MD, United States of America; 3 Division of Infectious Diseases, University of California, San Diego, CA (Currently Gilead Sciences, Foster City, CA), United States of America; 4 Department of Medicine, University of Pittsburgh, Pittsburgh, PA, United States of America; 5 Center for Biostatistics in AIDS Research, Harvard T.H. Chan School of Public Health, Boston, MA, United States of America; 6 Department of Molecular Biology and Microbiology, Tufts University, Boston, MA, United States of America; George Washington University, UNITED STATES

## Abstract

**Background:**

Identifying pre-ART factors associated with the emergence of HIV-1 drug resistance is critical for optimizing strategies to prevent virologic failure. A previous study reported that lower pre-ART HIV-1 *pol* diversity was associated with higher risk of virologic failure in HIV-1-infected children. To investigate this association in adults, we measured HIV-1 diversity with deep sequencing in pre-ART samples from adults with well-characterized virologic outcomes in a study (A5142) of initial ART conducted by the AIDS Clinical Trials Group (ACTG).

**Methods:**

We identified 22 cases in ACTG A5142 who experienced virologic failure with drug resistance mutations in RT and 44 matched controls who did not experience virologic failure. cDNA was synthesized from plasma HIV-1 RNA. Each cDNA molecule was tagged with a unique primer ID and RT codons 41–103 were amplified and deep sequenced. Sequences with the same tag were aligned and a consensus was generated to reduce PCR and sequencing errors. Diversity was calculated by measuring average pairwise distance (APD) of the consensus sequences. An exact conditional logistic regression model with percent APD as the risk factor estimated the odds ratio for VF and the corresponding 95% confidence interval.

**Results:**

Consensus single-genome sequences and diversity estimates of *pol* were obtained for pre-ART samples from 21 cases and 42 controls. The median (IQR) pre-ART percent APD was 0.71 (0.31–1.13) in cases and 0.58 (0.32–0.94) in controls. A possible trend was found for higher diversity being associated with greater risk of virologic failure in adults (OR = 2.2 per one percent APD increase, 95% CI = [0.8, 7.2]; p = 0.15).

**Conclusions:**

This study in adults suggests there is a positive association between higher pre-ART *pol* diversity and the risk of virologic failure in adults rather than an inverse relationship reported in children.

## Introduction

The emergence of HIV drug resistance is a barrier to sustained viral suppression from antiretroviral therapy (ART). HIV mutations conferring resistance to one or more antiretroviral drugs are often present in the viral population at low levels prior to initiating treatment [1–14]. This finding suggests that drug-resistant viral variants may be more likely to pre-exist in larger and more diverse viral populations. It is thought that virologic failure occurs when drug resistance mutations are linked on the same viral genomes allowing viral replication and spread of virus to new cells despite adherence to combination ART. The genetic diversity of HIV in plasma is typically very low after transmission [15–17] and increases at a rate of about 0.002% per day in *pro-pol* until reaching a plateau at about 3–5 years after infection [15]. It is likely that pre-existing drug resistance mutations, as described by Carr *et al*. [4] and Metzner *et al*. [8] using sensitive detection methods, are less frequent early after infection when the diversity of the viral population is low. Consequently, initiating ART soon after infection may reduce the risk of virologic failure. In contrast to this thesis, Chen *et al*. reported an inverse relationship between HIV *pol* diversity before ART and virologic failure in children; i.e. lower viral diversity was associated with greater risk of failure [18]. In that report, the investigators used melting temperatures to measure *pol* diversity in RNA or DNA samples collected at the baseline (prior to initiating ART) in infected children and found that there was a higher risk of virological failure in children with lower viral diversity compared to children with more diverse virus populations. It is possible that the cohort used for their analyses included children with higher incidence of transmitted drug resistance because the mothers had been exposed to either single-dose nevirapine or prior ART. After transmission of drug-resistant HIV, viral diversity could still be low but the risk of virologic failure would be higher than following transmission of wild-type virus, possibly explaining the association between low diversity and greater risk of failure in children. Furthermore, in some cases, sequencing of HIV DNA rather than plasma RNA, was used to calculate diversity. G to A hypermutants in HIV DNA that accumulate after transmission [19] may increase estimates of HIV diversity as compared to the diversity of the replicating viral populations in plasma.

To examine the relationship between HIV-1 diversity and risk of virologic failure in adults, we performed *pol-*targeted next-generation sequencing on pre-ART plasma samples from matched treatment-naive adults with and without virologic failure in the AIDS Clinical Trials Group (ACTG) A5142 study of initial ART [20]. We excluded sequences resulting from PCR resampling by tagging each cDNA molecule with a unique primer ID, similar to those described by Jabara *et al*. and Boltz, *et al*. [21] [22]. We then generated consensus sequences from the aligned reads containing identical primer IDs, reducing the error rate of next-generation sequencing as described [23]. After ensuring that the *sequences* were appropriately aligned and contained sufficient diversity signal to cluster only with the standard HIV genotype performed on the same pre-ART samples, we calculated the *pol* diversity in samples from each study participant prior to initiating ART. As described by Shao *et al*., we have developed methods to calculate HIV diversity of HIV populations from next generation sequencing data with the same accuracy as single-genome sequencing (the current gold standard for estimating intra-participant HIV diversity) by evaluating and accounting for errors introduced by the 454 platform [24]. After unblinding of study samples as to virologic outcome, we evaluated the relationship between intra-participant HIV *pol* diversity prior to initiating therapy and virologic failure on ART. We hypothesized that higher diversity in *pol* before ART initiation would have higher risk of virologic failure in adults.

## Methods

### Study population

Samples were obtained from ACTG trial A5142, a randomized trial comparing three drug combinations for initial ART: efavirenz plus two NRTIs (efavirenz group), lopinavir-ritonavir plus two NRTIs (lopinavir-ritonavir group), and lopinavir-ritonavir plus efavirenz (NRTI-sparing group) [20]. The study was approved by the institutional review board or ethics committee at each participating clinical site [20]. Each participant provided written informed consent. Consent was documented in each participant’s study source documents in accordance with DAIDS and local standards. The sites are regularly monitored by the DAIDS contract monitoring group (PPD) for compliance with the protocol and DAIDS policies. The participant characteristics of the A5142 study are described in detail in Riddler, et al. [20]. We included as cases all 22 participants randomized to the EFV+2 NRTI arm of A5142 who experienced virologic failure with drug resistance mutations in RT, and 44 matched controls from the same treatment arm who did not experience virologic failure. The participant characteristics used for matching of cases and controls are shown in Table 1, and those not used are shown in S1 Table. Controls were matched 2:1 to cases based on race/ethnicity, pre-ART CD4+ T-cell count within 50 cells/μl and pre-ART HIV-1 RNA within 0.5 log_10_ copies/mL (Table 1). One case was Asian, Pacific Islander, but did not have any matches due to the CD4+ T-cell count matching criterion, so was matched with 2 white non-Hispanic control participants. The CD4+ T-cell count and HIV-1 RNA matching criteria were widened to CD4+ T-cell count within 100 cells/μl and HIV-1 RNA within 1.0 log_10_ copies/mL for 1 case and to CD4+ T-cell count within 50 cells/μl and HIV-1 RNA within 1.0 log_10_ copies/mL for 3 other cases to have enough matches for each. Cases were matched with the 2 controls who had the closest pre-ART CD4+ T-cell count, one above and one below, when available.

**Table 1:** Pre-ART participants characteristics used for matching.

		Control	Case
		(N = 42)	(N = 21)
Race/ethnicity	White Non-Hispanic	6 (14%)	3 (14%)
	Black Non-Hispanic	24 (57%)	12 (57%)
	Hispanic (Regardless of Race)	12 (29%)	6 (29%)
HIV-1 RNA (log_10_(cps/mL))^†^	Mean (s.d.)	4.9 (0.6)	4.8 (0.8)
	Median (Q1-Q3)	4.8 (4.6–5.3)	4.8 (4.4–5.2)
	Min-Max	3.2–6.1	3.6–6.5
CD4+ T-cell count (cells/mm^3^)^†^	Mean (s.d.)	171 (157)	169 (159)
	Median (Q1-Q3)	118 (38–270)	140 (37–269)
	Min-Max	14–531	5–492

†Calculated as the mean of the last two measurments obtained prior to or at the start of ART or entry into trial.

Virologic failure was defined as a lack of suppression of plasma HIV-1 RNA by 1 log10 or rebound before week 32 or a lack of suppression to less than 200 copies per milliliter or rebound after week 32. HIV-1 drug resistance mutations in RT were defined by a modified 2008 IAS-USA list (https://www.iasusa.org/content/drug-resistance-mutations-in-HIV).

### Library preparation and next-generation sequencing

Pre-ART plasma samples from cases and controls were analyzed with blinding to virologic outcome. Plasma HIV RNA was extracted as described previously for single-genome sequencing [25] and cDNA was synthesized from 5000–10,000 copies of HIV-1 RNA, if enough copies were available. S2 Table shows the total number of HIV copies extracted from each sample according to the Roche Amplicor HIV Monitor assay, ultrasensitive version. Primer IDs were incorporated into the gene-specific primer used for cDNA synthesis along with a laboratory code to exclude cross contamination from samples run previously on the same instrument that may have been tagged with the same barcode (MID). The primer sequence used to synthesize the cDNA is shown in S1 Fig. Prior to cDNA synthesis, the extracted RNA was denatured at 82 degrees in the presence of 1mM dNTPs and 0.02μM gene-specific primer. cDNA was synthesized with Superscript III (Invitrogen) for one hour at 50 degrees and the RT enzyme was heat inactivated. The excess gene-specific primer was removed using 20 units of exonuclease I and 1 unit of FastAP Thermosensitive alkaline phosphatase (Thermo Scientific), which were then heat inactivated. A limited portion of *pol* (codons 41–103 of RT) was amplified from the tagged cDNA samples under real-time conditions to ensure high amplification efficiency. PCR primers included Roche designed 454 MIDs 1–36 (excluding MID 9) and catch probes for 454 Titanium sequencing (S1 Fig). Each forward PCR primer was used for two participants and the samples containing the same MID were identified by their location on the 454 sequencing plate. The same reverse PCR primer was used for each sample and is also shown in S1 Fig. PCR conditions were as follows: 95°C for 12 minutes followed by 3 cycles of 95°C for 10 seconds, 60°C for 10 seconds, and 72°C for 1 minute, then 45 cycles of 95°C for 15 seconds, 72°C for 1 minute. Following amplification, PCR products were gel-purified, quantified, pooled, and 454 Titanium sequencing was performed at the NCI core lab (Laboratory of Molecular Technology, Frederick, MD).

### Sequence analysis, quality control, and average pairwise diversity calculations

454 sequencing reads ≥335 bases in length were sorted into bins based on their MID after low quality reads (<Q20) were filtered out, and each read was mapped to an HIV-1 consensus B reference. An in-house Perl script was used to group the sequences in each bin that shared the identical primer IDs, and consensus sequences were generated for each group that had more than 5 sequencing reads. All consensus sequences from each participant were aligned to the baseline population sequences obtained by standard HIV genotyping (Quest Diagnostics) to ensure that the 454 sequences clustered with the appropriate sample population sequence. All consensus sequences from each of the participants were aligned and analyzed in a single quality control neighbor-joining p-distance tree in MEGA (http://www.megasoftware.net/) for two reasons: 1) to ensure that the fragment used for deep sequencing had appropriate signal to distinguish among participants and 2) to detect any genomes that were the result of sample or primer cross-contamination and exclude them from the analyses. The percent APDs of the aligned consensus sequences were calculated using PAPNC [24] with p-distance, a method that results in measurements of diversity from our alignments of 454 data with equal accuracy to single-genome sequencing [24]. For further quality assurance, we took steps to identify and omit PCR and/or sequencing errors in the primer IDs by generating distance trees of the primer IDs from samples where errors were likely (>30% of sequences containing primer IDs that were different by only a single base) and then determining a higher number of sequences required for the number of 454 reads to generate a consensus [26].

### Statistical analysis of average pairwise diversity calculations

Participant characteristics used for matching, as well as other pre-ART characteristics were summarized with descriptive statistics. The participant characteristics not used for matching were compared between cases and controls using the exact conditional logistic regression. An exact conditional logistic regression model with percent APD as the risk factor was used to estimate an odds ratio (OR) with corresponding 95% confidence interval (CI).

## Results

### Participant characteristics

The participant characteristics used for matching cases and controls are presented in Table 1, and those not used for matching are shown in S1 Table. Cases and controls were well matched for race/ethnicity, and pre-ART HIV plasma RNA levels and CD4+ T cells (Table 1). Despite not matching on age, sex, IV-drug use, or HIV subtype, these characteristics were well balanced between cases and controls (all p-values >0.26; S1 Table). Five of the participant samples had resistance mutations at baseline, three cases (PIDs 90307 M41L, 575667 M41L, and 397389 K103N) and two controls (508018 Y181C and 884697 V106M).

### Results from next-generation sequencing

The amplified *pol* fragments from each participant sample were deep sequenced by Roche 454 Titanium pyrosequencing in a single sequencing run. The 454 run resulted in 1,300,122 reads with >Q20 quality score and ≥335 bases in length. Intra-participant sequences with the same primer ID were used to generate consensus sequences, which represent single HIV genomes in the original sample. We used a cut off of 5 sequences with the same primer ID to generate a consensus. Ten of the 66 participant samples produced less than 20 consensus sequences. The primer binding regions for these samples were sequenced by bulk PCR amplification and Sanger sequencing. Nucleotide mismatches with the primers used for the generation of 454 libraries were identified in each case. New PCR primers were designed that matched the primer binding regions of the samples (S1 Fig) and 454 sequencing was repeated, resulting in 503,736 reads with >Q20 quality scores and ≥335 bases in length. After repeating the deep sequencing on these 20 samples (denoted in S2 Table with asterisks), 18 of 20 samples produced at least 20 consensus sequences, but two of the samples still failed to generate at least 20 consensus sequences and were excluded from analysis. After both 454 runs, a median of 258 consensus sequences per sample was obtained for the cases (range 47–655) and a median of 202 consensus sequences for the controls (range 25–1107). In total, one of the 22 cases and one of the two corresponding matched controls produced <20 consensus sequences even after the second 454 run; thus, both were excluded from analysis, along with the other matched control, leaving 21 evaluable cases, each with 2 matched controls for a total of 42 controls.

### Neighbor-joining analysis for quality control and assessment of HIV genetic signal

**Fig 1:**
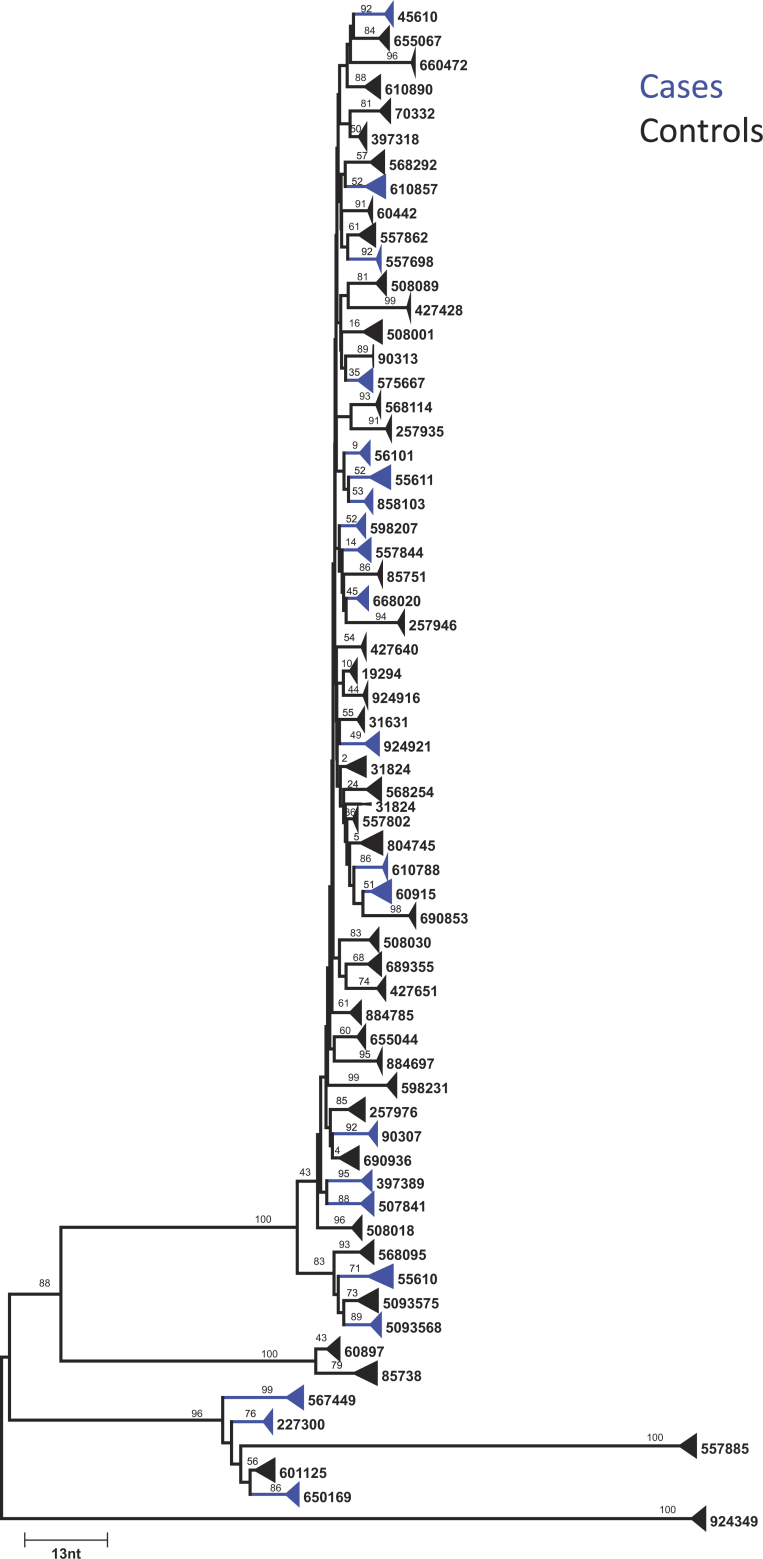
Neighbor-joining tree of 25 consensus sequences from each pre-ART participant sample using a requirement of 5 sequences containing the same primer ID to generate the consensus. Bootstrap values are shown for each branch. Cases are shown in blue and controls in black. The width of each triangle is indicative of the APD of HIV sequences in each participant.

As described in Methods, a distance tree was used to assess whether each set of 454 sequence data from a participant clustered appropriately with the standard HIV genotype for the same participant. Fig 1 shows a neighbor-joining, p-distance tree of 25 randomly selected consensus sequences generated from deep sequencing of samples from each participant in the cohort. The cases are shown in blue and the controls in black. The 454 consensus sequences from each participant clustered with the appropriate standard population genotype sequence except in one case for which a pre-ART population sequence was not available (PID 924916). This distance tree analysis showed that the region sequenced, despite being relatively short, had sufficient signal to accurately distinguish the viral populations from each participant.

### Identification of PCR/sequencing errors in primer IDs

PCR and sequencing errors can be introduced into primer IDs giving the false appearance of identical sequences derived from different templates when there was only one in the original RNA population [26]. By calculating the percent of primer IDs in each sample that were different from one another by single nucleotide changes, we identified 8 possible samples in our dataset in which errors in the primer IDs may have occurred at high rates (>30% of sequences), resulting in significant numbers of the same template being tagged with more than one primer ID. From these 8 participants, we generated neighbor-joining trees of the primer IDs and identified clusters that were different by only a single nucleotide change and contained the identical template sequence, confirming that the similar primer IDs were the result of PCR and/or sequencing error. To remove these artifacts from our dataset, we raised the cut off for the minimum number of sequences with the same primer ID to ≥20 for all samples. The difference in the number of consensus sequences and the percent APD obtained for each sample using a cut off of 20 vs. 5 is shown in S2 Table. The higher cut off resulted in two additional samples that now had fewer than 20 consensus sequences (the samples were from two controls corresponding to two different matched cases). These samples were not included in analysis with the higher cut off of 20 sequences for a consensus. These exclusions reduced the total number of participants to 21 cases and 40 controls (19 cases, each with 2 matched controls; and, 2 cases each with 1 matched control). APDs of cases and controls were calculated using both the 5 and 20 sequence requirements for a consensus sequence (S2 Table).

### Association between % Average Pairwise Difference (APD) and virologic failure

As above, HIV *pol* diversity was determined by APD for each pre-ART sample from the consensus sequences generated by 454 sequencing. After calculating the percent APD for each sample using a cut off of ≥5 sequences with the same primer ID to generate a consensus, the virologic outcome was unblinded and statistical analysis was performed to determine if *pol* diversity was associated with virologic failure. The median (IQR) percent APD of the pre-ART samples was 0.71 (0.31–1.13) for cases and 0.58 (0.32–0.94) for controls, revealing (Table 2) a possible trend for higher diversity being positively associated with greater risk of VF (OR = 2.2 per one percent APD increase, 95% CI = 0.8, 7.2), although this did not achieve significance (p = 0.15). When the required number of sequences to generate a consensus was raised to ≥20, the analysis confirmed a possible trend towards higher HIV *pol* diversity being associated with treatment failure (OR = 2.4 per one percent APD increase, 95% CI = [0.7, 8.9]; p = 0.15) (Table 2). Omitting the 4 participants that had drug resistance mutations detected in pre-ART samples did not change the outcome: the median APDs increased from 0.69% to 0.78% for the cases and from 0.49% to 0.53% for the controls. Individual measurements of APD at both cut offs (≥5 and ≥20) are shown in S2 Table. Overall, the results suggest that lower HIV diversity may not be associated with greater risk of VF in adults as has been reported for children [18].

**Table 2:** Percent Average Pairwise Distance (%APD) for each cut off.

Cut off*	% APD	Controls	Cases	Estimated OR per one %APD increase [95% Cl]	p-value
**≥ 5**	N	42	21		
	Mean (s.d.)	0.65 (0.43)	0.84 (0.59)	2.2 [0.8, 7.2]	0.15
	Median (Q1-Q3)	0.58 (0.32–0.94)	0.71 (0.31–1.13)		
	Min-Max	0.02–1.95	0.13–2.10		
**≥ 20**	N	40	21		
	Mean (s.d.)	0.58 (0.38)	0.76 (0.53)	2.4 [0.7, 8.9]	0.15
	Median (Q1-Q3)	0.49 (0.29–0.80)	0.69 (0.26–0.95)		
	Min-Max	0.02–1.76	0.11–2.06		

*Number of sequences with the same primer ID required to generate a consensus

## Discussion

We investigated the relationship between intra-participant HIV genetic diversity prior to initiating ART and the risk of virologic failure with HIV drug resistance on ART in adults. We hypothesized that adults having HIV populations with higher genetic diversity would have a greater probability of having pre-existing drug resistance mutations, and thus would be at increased risk of failure after initiating ART. Contrary to this hypothesis, a prior study by Chen *et al*. [18] reported an inverse relationship between pre-ART HIV diversity and ART failure in children. Their results suggested that either pre-existing drug resistance mutations emerge very early in children even when the overall viral diversity is low or that the study cohort may have included children with drug resistance mutations that were transmitted from the mother or acquired peripartum from nevirapine exposure. To test our hypothesis, we investigated the relationship between HIV diversity and treatment outcome in a cohort of adults by estimating *pol* APD in blinded, pre-ART plasma samples from participants with well-characterized virologic outcomes in the A5142 study of initial ART [20].

In our study, HIV-1 diversity in the cases (those participants experiencing virologic failure) and matched controls (those who did not) revealed a possible trend (p = 0.15) towards higher HIV *pol* genetic diversity being associated with virologic failure in adults. Although suggestive, our results do not reject the null hypothesis that there is no association between high diversity and ART failure. Nevertheless, our results are not consistent with those found in children by Chen et al. [18] of a *negative* correlation between HIV diversity and ART failure. The likely explanation for these apparently opposing findings is that Chen *et al*. studied the relationship between HIV diversity and virologic failure in children who acquired HIV through maternal transmission. The mother and babies, in many cases, had been exposed to nevirapine or ART to prevent mother to child transmission. As a consequence, the children may have acquired HIV drug resistance mutations from maternal to children transmission or early after birth from peripartum nevirapine exposure. Such transmitted or acquired resistance could result in HIV populations of low diversity being associated with virologic failure with subsequent ART and would explain the different results in children and adults.

Rather than using heteroduplex melting temperatures to estimate diversity as in Chen, *et al*. [1], we utilized Roche 454 next generation sequencing. In generating our libraries for deep sequencing, we tagged each cDNA molecule with a primer ID, similar to those described by Jabara *et al*. [21]. The primer ID strategy allowed us to overcome several important issues associated with 454 sequencing: 1) we were able to significantly reduce PCR and sequencing error since multiple sequencing reads derived from the same template could be aligned and a consensus sequence generated that more accurately reflects the original template, and 2) we were able to eliminate PCR resampling (i.e., multiple amplicons derived from one template) because we could identify and segregate amplicons that originated from the same cDNA molecule. Nevertheless, other issues are introduced by incorporating primer IDs such as chance use of the same primer ID on more than one template in a sample (i.e., the birthday effect), and PCR errors introduced into the primer IDs resulting in the same template being tagged with non-identical primer IDs. We were able to exclude these artifacts from our dataset by taking several steps. First, we started with only 5000 copies of HIV RNA from each participant sample and used primer IDs containing ten random nucleotides, resulting in >10^6^ different primer ID possibilities, which is 200-fold higher than the number of RNA templates. If all 5000 sequences are amplified, the probability of the same primer ID being used twice by chance is (1-4^-10^)^5000^ or about 0.005. Moreover, in the event that, even with this low probability, two identical primer IDs did label two different RNA molecules, the less frequent of these would have been excluded from our analyses by generating the consensus sequence. To address the issue of PCR errors introduced into primer IDs resulting in the original template being tagged with different primer IDs, we analyzed all the primer IDs in the data set of the consensus sequences to identify those that were different from each other by only a single nucleotide, indicating that they may have arisen from PCR error. We identified samples (N = 8) in which single nucleotide differences in primer IDs were common when only 5 sequences were required to generate a consensus. Therefore, we reanalyzed the data set after increasing the number of sequences required to generate a consensus to >20, which eliminated sequences that were tagged with more than one primer IDs from the analysis. By taking these quality control steps, we were able to leverage the advantages of primer IDs without sacrificing the quality of our dataset from problems that could have been associated with their use.

We previously demonstrated that diversity in *pro-pol* typically increases in the early years of infection at a consistent rate of about 0.002% per day [15], making this genomic region useful for estimating the duration of infection after transmission of a single viral variant. The consistent rate of mutations in *pro-pol* also suggests that overall HIV diversity will be associated with the frequency of rare, drug-resistant variants [27]. The possible trend found in the current study between diversity and virologic failure lends support for this thesis and suggests that adults treated in chronic infection, when viral diversity has accumulated, are at higher risk of virologic failure compared with those treated early after infection, although further investigation is warranted. Importantly, our results are different from those of Chen, *et al*. [18], and suggest that low diversity in adults may not associated with virologic failure as it is in children. The implication from our study is initiating ART early after HIV infection in adults before viral diversity accumulates could reduce the risk of virologic failure on ART, which further supports current recommendations to initiate ART without delay in all persons with HIV infection.

## Supporting information

S1 Fig Sequences of all primers used for cDNA synthesis and for PCR amplification. The participant IDs associated with each primer are also shown. The subregions comprising each primer are color coded as indicated.
(PDF)

S1 Table Pre-ART participant characteristics NOT used for matching. Despite not matching on age, sex, IV-drug use, or HIV subtype, these characteristics were well balanced between cases and controls (all p-values >0.26).
(DOCX)

S2 Table Plasma HIV diversity at pre-ART. Average pairwise distances were slightly higher in participants who experienced virologic failure (cases) compared to those who did not fail ART (controls).
(DOCX)
