# Supplementary material for: Lower pre-ART intra-participant HIV-1 pol diversity may not be associated with virologic failure in adults
Source: PLoS One. 2018 Jan 25;13(1):e0190438. doi: 10.1371/journal.pone.0190438 (PMC5784902; doi:10.1371/journal.pone.0190438)
Supplement: S1 Fig — The participant IDs associated with each primer are also shown. The subregions comprising each primer are color coded as indicated. (PDF) [file pone.0190438.s001.pdf]

Primers used for 454 sequencing

	Unique Lab Code	Primer ID	Complimentary HIV Sequence
cDNA primer	GGTATCGAAGTCATCCTGCTAG	NNNNNNNNNN	TTGCTGGTGATCCTTTCCATCC

	Complimentary HIV Sequence	454 Key	Unique Lab Code
PCR reverse primers	CCTATCCCCTGTGTGCCTTGGCAGTC	TCAG	GGTATCGAAGTCATCCTGCTAG

Forward Primers

PID	454 Primer A	MID	HIV Sequence
655067	CCATCTCATCCCTGCGTGTCTCCGACTCAG	ACGAGTGCGT	GCCTGAAAATCCATACAATACTCC
55611, 655044	CCATCTCATCCCTGCGTGTCTCCGACTCAG	ACGCTCGACA	GCCTGAAAATCCATACAATACTCC
56101, 689355	CCATCTCATCCCTGCGTGTCTCCGACTCAG	AGACGCACTC	GCCTGAAAATCCATACAATACTCC
60442, 610890	CCATCTCATCCCTGCGTGTCTCCGACTCAG	AGCACTGTAG	GCCTGAAAATCCATACAATACTCC
60915	CCATCTCATCCCTGCGTGTCTCCGACTCAG	ATCAGACACG	GCCTGAAAATCCATACAATACTCC
60897, 610788	CCATCTCATCCCTGCGTGTCTCCGACTCAG	ATATCGCGAG	GCCTGAAAATCCATACAATACTCC
31631, 668020	CCATCTCATCCCTGCGTGTCTCCGACTCAG	CGTGTCTCTA	GCCTGAAAATCCATACAATACTCC
31824, 660472	CCATCTCATCCCTGCGTGTCTCCGACTCAG	CTCGCGTGTC	GCCTGAAAATCCATACAATACTCC
90313, 690936	CCATCTCATCCCTGCGTGTCTCCGACTCAG	TGATACGTCT	GCCTGAAAATCCATACAATACTCC
85751, 690853	CCATCTCATCCCTGCGTGTCTCCGACTCAG	CATAGTAGTG	GCCTGAAAATCCATACAATACTCC
85738, 650169	CCATCTCATCCCTGCGTGTCTCCGACTCAG	CGAGAGATAC	GCCTGAAAATCCATACAATACTCC
45610, 257976	CCATCTCATCCCTGCGTGTCTCCGACTCAG	ATACGACGTA	GCCTGAAAATCCATACAATACTCC
19294, 257935	CCATCTCATCCCTGCGTGTCTCCGACTCAG	TCACGTACTA	GCCTGAAAATCCATACAATACTCC
70332, 257946	CCATCTCATCCCTGCGTGTCTCCGACTCAG	CGTCTAGTAC	GCCTGAAAATCCATACAATACTCC
575667	CCATCTCATCCCTGCGTGTCTCCGACTCAG	TCTACGTAGC	GCCTGAAAATCCATACAATACTCC
507841, 227300	CCATCTCATCCCTGCGTGTCTCCGACTCAG	TGTACTACTC	GCCTGAAAATCCATACAATACTCC
508018, 397318	CCATCTCATCCCTGCGTGTCTCCGACTCAG	ACGACTACAG	GCCTGAAAATCCATACAATACTCC
508089, 397389	CCATCTCATCCCTGCGTGTCTCCGACTCAG	CGTAGACTAG	GCCTGAAAATCCATACAATACTCC
508001	CCATCTCATCCCTGCGTGTCTCCGACTCAG	TACGAGTATG	GCCTGAAAATCCATACAATACTCC
508030, 924349	CCATCTCATCCCTGCGTGTCTCCGACTCAG	TACTCTCGTG	GCCTGAAAATCCATACAATACTCC
557885, 924921	CCATCTCATCCCTGCGTGTCTCCGACTCAG	TAGAGACGAG	GCCTGAAAATCCATACAATACTCC
557802, 924916	CCATCTCATCCCTGCGTGTCTCCGACTCAG	TCGTCGCTCG	GCCTGAAAATCCATACAATACTCC
557844, 804745	CCATCTCATCCCTGCGTGTCTCCGACTCAG	ACATACGCGT	GCCTGAAAATCCATACAATACTCC
557862	CCATCTCATCCCTGCGTGTCTCCGACTCAG	ACGCGAGTAT	GCCTGAAAATCCATACAATACTCC
557698, 858103	CCATCTCATCCCTGCGTGTCTCCGACTCAG	ACTACTATGT	GCCTGAAAATCCATACAATACTCC
884785	CCATCTCATCCCTGCGTGTCTCCGACTCAG	ACTGTACAGT	GCCTGAAAATCCATACAATACTCC
568292, 884697	CCATCTCATCCCTGCGTGTCTCCGACTCAG	AGACTATACT	GCCTGAAAATCCATACAATACTCC
568254, 427640	CCATCTCATCCCTGCGTGTCTCCGACTCAG	AGCGTCGTCT	GCCTGAAAATCCATACAATACTCC
427651	CCATCTCATCCCTGCGTGTCTCCGACTCAG	AGTACGCTAT	GCCTGAAAATCCATACAATACTCC
568114, 427428	CCATCTCATCCCTGCGTGTCTCCGACTCAG	ATAGAGTACT	GCCTGAAAATCCATACAATACTCC
598207	CCATCTCATCCCTGCGTGTCTCCGACTCAG	CACGCTACGT	GCCTGAAAATCCATACAATACTCC
598231, 5093575	CCATCTCATCCCTGCGTGTCTCCGACTCAG	CAGTAGACGT	GCCTGAAAATCCATACAATACTCC
601125	CCATCTCATCCCTGCGTGTCTCCGACTCAG	CGACGTGACT	GCCTGAAAATCCATACAATACTCC

Samples repeated where there were primer mismatches:

PID			
55610	CCATCTCATCCCTGCGTGTCTCCGACTCAG	ACGCTCGACA	GCCTGAAAATCCATATAACACTCC
557698	CCATCTCATCCCTGCGTGTCTCCGACTCAG	AGACGCACTC	GCCTGAAAATCCATATAACACTCC
568095	CCATCTCATCCCTGCGTGTCTCCGACTCAG	AGCACTGTAG	GCCTGAAAATCCATATAACACTCC
5093575	CCATCTCATCCCTGCGTGTCTCCGACTCAG	CGTGTCTCTA	GCCTGAAAATCCATATAACACTCC
5093568	CCATCTCATCCCTGCGTGTCTCCGACTCAG	CATAGTAGTG	GCCTGAAAATCCATATAACACTCC
90307	CCATCTCATCCCTGCGTGTCTCCGACTCAG	CGAGAGATAC	GCCTGAAAATCCATATAATACTCC
598231	CCATCTCATCCCTGCGTGTCTCCGACTCAG	ATACGACGTA	GCCTGAAAATCCATACAATACTMC
610857	CCATCTCATCCCTGCGTGTCTCCGACTCAG	TGTACTACTC	GCCTGAAAATCCATATAAYACTCC
