# Supplementary material for: Lower pre-ART intra-participant HIV-1 pol diversity may not be associated with virologic failure in adults
Source: PLoS One. 2018 Jan 25;13(1):e0190438. doi: 10.1371/journal.pone.0190438 (PMC5784902; doi:10.1371/journal.pone.0190438)
Supplement: S1 Table — Despite not matching on age, sex, IV-drug use, or HIV subtype, these characteristics were well balanced between cases and controls (all p-values >0.26). (DOCX) [file pone.0190438.s002.docx]

S1 Table: Pre-ART Participant Characteristics NOT Used for Matching

|  | | **Control** | **Case** |
| --- | --- | --- | --- |
|  |  | **(N=42)** | **(N=21)** |
| Sex^†^ | Male | 32 (76%) | 16 (76%) |
|  | Female | 10 (24%) | 5 (24%) |
| Age (years)^†^ | Mean (s.d.) | 37 (8) | 36 (7) |
|  | Median (Q1-Q3) | 37 (32-42) | 37 (32-40) |
|  | Min-Max | 21-55 | 24-48 |
| RT Consensus Subtype^†^ | B | 40 (95%) | 18 (86%) |
|  | C | 2 (5%) | 3 (14%) |
| IV Drug Use^†^ | Never | 37 (88%) | 21 (100%) |
|  | Previously/currently | 5 (12%) | 0 (0%) |

*† Exact conditional logistic regression: all p-values > 0.26*
