# Supplementary material for: Lower pre-ART intra-participant HIV-1 pol diversity may not be associated with virologic failure in adults
Source: PLoS One. 2018 Jan 25;13(1):e0190438. doi: 10.1371/journal.pone.0190438 (PMC5784902; doi:10.1371/journal.pone.0190438)
Supplement: S2 Table — Average pairwise distances were slightly higher in participants who experienced virologic failure (cases) compared to those who did not fail ART (controls). (DOCX) [file pone.0190438.s003.docx]

S2 Table. Plasma HIV diversity at pre-ART

|  | | | | **cut off >5†** | | | **cut off >20†** | | |
| --- | --- | --- | --- | --- | --- | --- | --- | --- | --- |
| **Cases** | **Patient ID** | **HIV RNA**  **copies/ml** | **# HIV RNA**  **copies** | **# of**  **consensus** | **% APD** | **% Standard**  **Error** | **# of consensus**  **sequences** | **% APD** | **% Standard**  **Error** |
|  | *55610 | 4466433 | 10000 | 181 | 2.1 | 0.007 | 151 | 2.06 | 0.008 |
|  | 55611 | 21998 | 10000 | 244 | 1.86 | 0.005 | 180 | 1.79 | 0.006 |
|  | 56101 | 269180 | 10000 | 292 | 0.96 | 0.003 | 226 | 0.95 | 0.003 |
|  | 60915 | 379369 | 10000 | 507 | 0.5 | 0.001 | 338 | 0.49 | 0.002 |
|  | *90307 | 5172 | 2586 | 47 | 0.7 | 0.016 | 20 | 0.63 | 0.033 |
|  | 45610 | 25214 | 10000 | 435 | 0.24 | 0.001 | 270 | 0.25 | 0.002 |
|  | 575667 | 48252 | 10000 | 163 | 0.28 | 0.003 | 137 | 0.26 | 0.003 |
|  | 507841 | 695865 | 10000 | 266 | 0.36 | 0.002 | 204 | 0.3 | 0.003 |
|  | 557844 | 99068 | 10000 | 497 | 1.25 | 0.002 | 229 | 1.17 | 0.004 |
|  | *557698 | 528027 | 10000 | 258 | 0.13 | 0.001 | 204 | 0.11 | 0.002 |
|  | 567449 | 15579 | 7789 | 245 | 1.13 | 0.004 | 212 | 1.05 | 0.004 |
|  | 598207 | 58532 | 10000 | 259 | 0.31 | 0.002 | 227 | 0.25 | 0.002 |
|  | *610857 | 6377 | 3189 | 52 | 1.35 | 0.02 | 37 | 1.43 | 0.027 |
|  | 610788 | 28346 | 10000 | 655 | 0.16 | 0.001 | 318 | 0.14 | 0.001 |
|  | 668020 | 83087 | 10000 | 144 | 0.92 | 0.005 | 128 | 0.89 | 0.006 |
|  | 650169 | 34069 | 10000 | 551 | 0.64 | 0.001 | 330 | 0.62 | 0.002 |
|  | 227300 | 7893 | 3947 | 293 | 0.22 | 0.002 | 200 | 0.25 | 0.002 |
|  | 397389 | 71163 | 10000 | 110 | 1.81 | 0.029 | 91 | 0.76 | 0.007 |
|  | 924921 | 81671 | 10000 | 563 | 0.98 | 0.002 | 340 | 0.87 | 0.002 |
|  | 858103 | 2802 | 1401 | 83 | 0.71 | 0.009 | 51 | 0.69 | 0.014 |
|  | *5093568 | 525000 | 10000 | 196 | 0.97 | 0.004 | 169 | 0.94 | 0.005 |
|  | **median** | **58532** | **10000** | **258** | **0.71** | **0.003** | **203** | **0.69** | **0.003** |
| **Controls** | 60442 | 53421 | 10000 | 164 | 0.26 | 0.002 | 144 | 0.26 | 0.003 |
|  | 60897 | 5759 | 2880 | 26 | 1.11 | 0.051 | 18 | - | - |
|  | 31631 | 69827 | 10000 | 125 | 0.61 | 0.006 | 110 | 0.54 | 0.005 |
|  | 31824 | 491429 | 10000 | 25 | 1.95 | 0.062 | 11 | - | - |
|  | 90313 | 22069 | 10000 | 385 | 0.02 | 0.000 | 317 | 0.02 | 0.000 |
|  | 85751 | 592453 | 10000 | 158 | 0.21 | 0.003 | 129 | 0.16 | 0.003 |
|  | 85738 | 52997 | 10000 | 427 | 1.37 | 0.002 | 280 | 1.3 | 0.004 |
|  | 19294 | 234985 | 10000 | 158 | 0.49 | 0.003 | 125 | 0.46 | 0.004 |
|  | 70332 | 53562 | 10000 | 251 | 0.61 | 0.002 | 220 | 0.62 | 0.003 |
|  | 508018 | 69187 | 10000 | 697 | 0.2 | 0.001 | 198 | 0.11 | 0.002 |
|  | 508089 | 48630 | 10000 | 738 | 0.66 | 0.001 | 454 | 0.61 | 0.002 |
|  | 508001 | 346471 | 10000 | 240 | 1.05 | 0.003 | 210 | 1.04 | 0.004 |
|  | 508030 | 337908 | 10000 | 281 | 0.94 | 0.003 | 186 | 0.91 | 0.004 |
|  | 557885 | 22802 | 10000 | 97 | 0.32 | 0.004 | 70 | 0.31 | 0.006 |
|  | 557802 | 22015 | 10000 | 196 | 0.42 | 0.003 | 165 | 0.41 | 0.004 |
|  | 557862 | 28041 | 10000 | 106 | 0.83 | 0.007 | 100 | 0.78 | 0.007 |
|  | 568292 | 30213 | 10000 | 324 | 1.73 | 0.003 | 271 | 1.76 | 0.004 |
|  | 568254 | 892377 | 10000 | 143 | 0.55 | 0.004 | 121 | 0.52 | 0.004 |
|  | *568095 | 454421 | 10000 | 327 | 1.06 | 0.003 | 286 | 1.06 | 0.003 |
|  | 568114 | 31667 | 10000 | 116 | 0.27 | 0.004 | 103 | 0.27 | 0.004 |
|  | *598231 | 37072 | 10000 | 25 | 0.62 | 0.027 | 22 | 0.63 | 0.030 |
|  | 601125 | 27096 | 10000 | 702 | 0.68 | 0.001 | 427 | 0.61 | 0.002 |
|  | 655067 | 51151 | 10000 | 64 | 0.39 | 0.01 | 41 | 0.31 | 0.012 |
|  | 655044 | 488372 | 10000 | 314 | 0.22 | 0.001 | 226 | 0.21 | 0.002 |
|  | 689355 | 1973 | 987 | 49 | 0.78 | 0.016 | 38 | 0.81 | 0.020 |
|  | 610890 | 160367 | 10000 | 735 | 1.34 | 0.001 | 271 | 1.26 | 0.004 |
|  | 660472 | 36576 | 10000 | 494 | 0.22 | 0.001 | 363 | 0.17 | 0.001 |
|  | 690936 | 474326 | 10000 | 73 | 0.98 | 0.012 | 56 | 1.02 | 0.016 |
|  | 690853 | 58421 | 10000 | 1107 | 0.51 | 0.000 | 381 | 0.44 | 0.001 |
|  | 257976 | 45464 | 10000 | 362 | 0.68 | 0.002 | 277 | 0.66 | 0.002 |
|  | 257935 | 1078054 | 10000 | 161 | 0.23 | 0.003 | 128 | 0.22 | 0.003 |
|  | 257946 | 5169 | 2585 | 98 | 0.3 | 0.005 | 68 | 0.25 | 0.006 |
|  | 397318 | 37839 | 10000 | 71 | 0.32 | 0.008 | 63 | 0.31 | 0.008 |
|  | 924349 | 37293 | 10000 | 290 | 0.65 | 0.002 | 228 | 0.64 | 0.003 |
|  | 924916 | 534215 | 10000 | 253 | 0.33 | 0.002 | 222 | 0.32 | 0.002 |
|  | 804745 | 83663 | 10000 | 559 | 1.13 | 0.002 | 437 | 1.08 | 0.002 |
|  | 884785 | 31434 | 10000 | 235 | 0.43 | 0.003 | 195 | 0.42 | 0.003 |
|  | 884697 | 838156 | 10000 | 197 | 0.4 | 0.003 | 161 | 0.4 | 0.003 |
|  | 427640 | 45603 | 10000 | 751 | 0.25 | 0.001 | 279 | 0.13 | 0.001 |
|  | 427651 | 1142574 | 10000 | 163 | 0.74 | 0.004 | 146 | 0.73 | 0.005 |
|  | 427428 | 80665 | 10000 | 206 | 0.36 | 0.002 | 181 | 0.35 | 0.003 |
|  | *5093575 | 156000 | 10000 | 90 | 0.96 | 0.012 | 79 | 0.93 | 0.013 |
|  | **median** | **53492** | **10000** | **202** | **0.58** | **0.003** | **173** | **0.49** | **0.003** |

*Insufficient sequences obtained in original 454 run. A plasma sample from the same time point was rerun with new primers.

†Number of sequences with the same primer ID required to generate a consensus.
